# The Replicase Protein of Potato Virus X Is Able to Recognize and *Trans-*Replicate Its RNA Component

**DOI:** 10.3390/v16101611

**Published:** 2024-10-15

**Authors:** Pinky Dutta, Andres Lõhmus, Tero Ahola, Kristiina Mäkinen

**Affiliations:** 1 Department of Agricultural Sciences, Faculty of Agriculture and Forestry, University of Helsinki, 00014 Helsinki, Finland; pinky.dutta@helsinki.fi; 2 Department of Microbiology, Faculty of Agriculture and Forestry, University of Helsinki, 00014 Helsinki, Finland; andres.lohmus@gmail.com (A.L.); tero.ahola@helsinki.fi (T.A.)

**Keywords:** *Alphaflexiviridae*, RNA virus, *Potexvirus*, PVX, RNA-dependent RNA polymerase

## Abstract

The *trans-*replication system explores the concept of separating the viral RNA involved in the translation of the replicase protein from the replication of the viral genome and has been successfully used to study the replication mechanisms of alphaviruses. We tested the feasibility of this system with potato virus X (PVX), an alpha-like virus, *in planta*. A viral RNA template was designed which does not produce the replicase and prevents virion formation but remains recognizable by the replicase. The replicase construct encodes for the replicase protein, while lacking other virus-specific recognition sequences. Both the constructs were delivered into *Nicotiana benthamiana* leaves via *Agrobacterium*-mediated infiltration. Templates of various lengths were tested, with the longer templates not replicating at 4 and 6 days post inoculation, when the replicase protein was provided *in trans*. Co-expression of helper component proteinase with the short template led to its *trans-*replication. The cells where replication had been initiated were observed to be scattered across the leaf lamina. This study established that PVX is capable of *trans*-replicating and can likely be further optimized, and that the experimental freedom offered by the system can be utilized to delve deeper into understanding the replication mechanism of the virus.

## 1. Introduction

Potato virus X (PVX; genus *Potexvirus*; family *Alphaflexiviridae*) is one of the most studied plant viruses [1]. The virion is 470–580 nm in length and 12–13 nm in diameter, non-enveloped, filamentous, and flexuous. The viral RNA (vRNA), which serves both as the genome and the viral messenger RNA (mRNA), is linear, (+)-stranded, and encapsidated within 1300 coat protein (CP) subunits in a helical manner. A methylated cap and a poly-A tail border the 6.4-kb PVX genome at its 5′- and 3′-terminals, respectively [2]. The viral genome encodes for five open reading frames (ORFs) [3]. The first ORF encodes the 165 kDa replicase, which is produced from the genomic RNA (gRNA) and is the only protein known to be essential for the replication of PVX [4,5,6]. Its coding region has three domains, namely methyltransferase, helicase, and RNA-dependent RNA polymerase [7], with all three domains being important for the protein to be functional. During infection, the replicase is seen to localize to the endoplasmic reticulum (ER)-derived membrane structures along with TGBp3, but the mechanism of membrane association is not known [5,8]. The second, third, and fourth ORFs encode for a cluster of three overlapping genes, collectively known as the “triple block genes” (TGBs), which are translated into three proteins (TGBp1, TGBp2, and TGBp3). Two of these proteins (TGBp1 and TGBp2) are produced from two separate sgRNAs, while TGBp3 is produced by leaky scanning of the TGB2 sub-genomic RNA (sgRNA)s. The TGBp1 or p25 is involved in vRNA transport and translation, has anti-viral silencing suppressor activity [9], and is also the main pathogenicity determinant of PVX responsible for symptom development and potex-potyviral synergistic interaction [10]. TGBp2 and TGBp3 associate with the ER and have transmembrane domains [11,12]. TGBp2 triggers the formation of ER membrane-derived vesicles, which are essential for viral movement [13]. A 25 kDa CP is encoded by the last ORF, which is also produced from a sub-genomic (sg) promoter [3,14]. It is involved in the formation of virions, both short distance cell-to-cell movement and long distance vascular transport, and also acts as an elicitor of R-gene-mediated resistance in plants [15]. The origin of assembly sequence in PVX lies in the 5′-untranslated region (UTR) and overlaps with sequences important for viral replication and movement [16,17,18]. In PVX, only gRNA is reported to be encapsidated; however, in other potexviruses, such as narcissus mosaic virus and bamboo mosaic virus, sgRNA is also packed [19,20,21].

The coding sequence of PVX is flanked at its 5′- end by an 84 nt UTR [22] and by a 74 nt 3′-UTR [23]. These *cis*-acting sequences are highly conserved amongst potexviruses and are essential for viral replication [24] and movement [18]. Two stem loop structures are formed from the 5′-UTR which also include sequences from the first ORF. The first stem loop, 5′SL1 (32–106 nt), is versatile and is involved in the synthesis of (+) strand RNA and also regulates sgRNA levels [25,26]. Any mutation in the octa-nucleotide sequence (AACUAAAC) present in the 5′-UTR, reduces complementarity with sgRNA and lowers generation of gRNA and sgRNA [27]. The UTR at the 3′- end contains three stem loop structures, 3′SL1, 3′SL2, and 3′SL3 [23]. The 3′SL3, along with a hexanucleotide sequence (ACAUAA) present at terminal of the 3′SL2 and the poly-A tail, is essential for the synthesis and accumulation of minus strand RNA [23,28]; the near upstream elements (NUEs) in the 3′-UTR, NUE1, and NUE2 are vital for accumulation of (+) strand RNA [29,30]. Deletion of the NUEs could lead to a decrease in polyadenylation of the RNA, thus rendering it unstable. Host factors, such as chloroplast phosphoglycerate kinase (p43) and p51, bind to a U-rich sequence (UAUUUUCU) present in the 3′ UTR which is needed for the accumulation of (-) strand RNA [31] and viral movement [32]. Similarly to the conserved sequences in the 5′UTR, the interaction of a hexanucleotide sequence in the 3′SL3 with the sg promoter is necessary for minus strand synthesis [33].

The *trans-*replication system has been implemented to study the biology of several alphaviruses [34,35] It principally relies on uncoupling the production of the replication machinery from genome replication. In practice, the replication protein(s) are expressed from a separate non-replicating mRNA. Our study was conducted to test for the feasibility of the *trans-*replication system with PVX. The objective was to generate a replicase expression construct and several template expression constructs and agroinfiltrate them into *Nicotiana benthamiana* leaves to investigate if PVX could replicate *in trans*. Although a significant amount of work has already been conducted to study PVX biology, the trans-replication system would allow greater flexibility in manipulating the viral replicase protein and grant a deeper understanding into PVX’s replication mechanism and cell biology. Our results show that a shorter template can replicate in individual cells when replicase is expressed *in trans*. The expression of anti-viral silencing suppressors together with the short template and the replicase can further help to sustain the *trans*-replication system in *N. benthamiana.*

## 2. Materials and Methods

### 2.1. Molecular Cloning

#### 2.1.1. The Replicase Expression Construct

The coding sequence of the replicase protein was generated by gradient PCR, using a forward primer (5′-ATGGCCAAGGTGCG-3′) and a reverse primer (5′-TTAAAGAAAGTTTCTGAGGCG-3′) and an infectious cDNA clone of PVX tagged with *Renilla luciferase* reporter gene PVX-Rluc [36] as the PCR template; the pGR106 vector backbone [37], along with the 35S promoter sequence and nopaline synthase (*nos*) terminator sequence, was obtained using a forward and reverse primer pair (5′-AGAAACTTTCTTTAACTAGTGGGTACCGCGAATTTCC-3′ and 5′-GCGCACCTTGGCCATTCCTCTCCAAATGAAATGAACTTCC-3′) in a 20 μL reaction volume containing 50 ng of pGR106. Similar PCR products were combined and purified using the GeneJET PCR Purification Kit (Thermo Fisher Scientific, Lithuania). The PVX replicase construct was assembled using a NEBuilder HiFi DNA Assembly Cloning Kit (New England Biolabs Inc., USA) according to the manufacturer’s instructions. Plasmids from single *Escherichia coli* colonies were extracted using the alkaline lysis method and were screened using *Kpn*I restriction enzyme (New England Biolabs Inc.). The positive plasmid was used as a template in a PCR reaction with 5′-ACCCTCGAGAATTCGAGCTCGGTACCATGGCCAAGGTGCGC-3′ and 5′-CCACTCGAGTCCTCTCCAAATGAAATGAACTTCC-3′ as forward and reverse primers, respectively, [38] with an *Xho*I restriction site (underlined) immediately before the replicase start codon. This was set up to engineer a reference leader sequence in the replicase construct [38]. The resultant PCR fragment was treated with *Dpn*I and *Xho*I and run on a 0.75% agarose gel to purify and extract the desired fragment using a GeneJET Gel Extraction Kit (Thermo Fisher Scientific). The purified fragment obtained was ligated by incubating for 24 h at 4 °C using T4 DNA ligase (New England Biolabs Inc.). The ligation mix was then transformed into competent DH5α *E. coli* cells. The plasmids obtained from the single *Escherichia coli* cells were verified using *Hind*III and *Xho*I restriction enzymes. 

The desired icDNA replicase construct, and all the following constructs described below, were transformed into competent *Agrobacterium tumefaciens GV3101* strain containing the helper plasmid pSoup [39] via electroporation. An infectious cDNA clone of PVX tagged with green fluorescent protein (GFP), PVX-GFP [36], was also used as an additional replicase control in some of the experiments, as detailed in the figure legends.

#### 2.1.2. Template Constructs

All template constructs were created with either the introduction of frameshift mutations or deletions to PVX-Rluc.

##### Introduction of Frameshift Mutation

**Figure 1:**
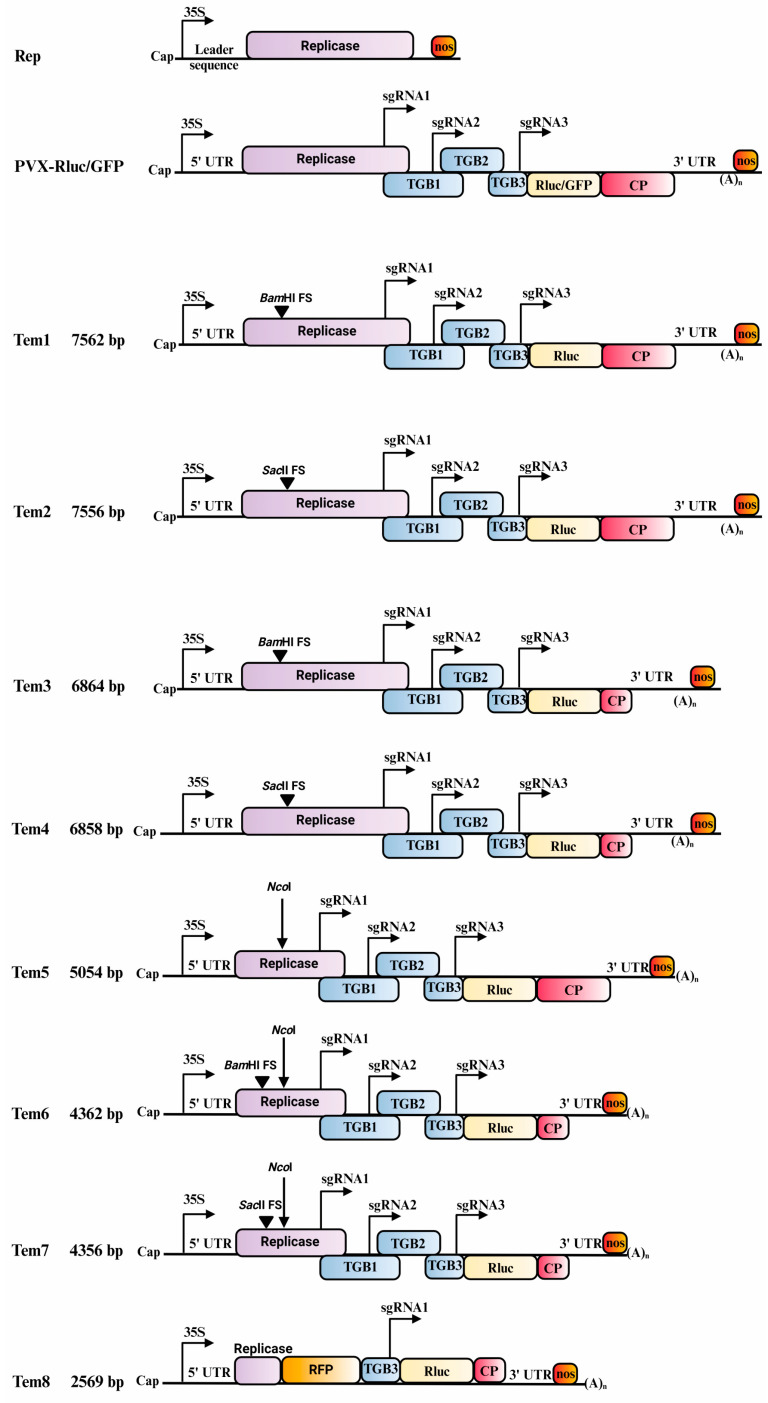
Schematic representation of the replicase, PVX and template constructs. The constructs are not drawn to scale.

Restriction digestion with *Bam*HI or *Sac*II was conducted independently on PVX-Rluc to introduce a frameshift mutation at the coding sequence of the replicase protein. The DNA ends were filled in using T4 DNA polymerase (New England Biolabs Inc.) according to the manufacturer’s instructions and re-ligated using T4 DNA ligase (New England Biolabs Inc.), before being transformed into competent DH5α *E. coli* cells. The positive plasmids were screened using *Sac*II/*Bam*HI and *Stu*I restriction enzymes. These constructs were named Tem1 and Tem2 (Figure 1).

##### Deletion of Coat Protein Coding Sequence

Restriction digestion with *Sal*I and *Xho*I was performed on the PVX-Rluc frameshift genome to delete the coding sequence of the viral CP, leaving behind only 60 base pairs (bp) of the CP sequence. The purified plasmid fragments were filled in using T4 DNA polymerase (New England Biolabs Inc.) and were ligated. The ligation mix was later transformed into DH5α cells and screened for the positive colonies using *Eco*RI and *Hpa*I restriction enzymes. These constructs were named Tem3 and Tem4 (Figure 1).

##### Medium and Short Templates

An *Nco*I (New England Biolabs Inc.) restriction digestion was performed on PVX-Rluc, Tem3, and Tem4 templates to remove 2502 nucleotides from the replicase sequence to yield shorter length templates of 5054, 4362, and 4364 nucleotides. These constructs were named Tem5, Tem6, and Tem7, respectively.

An even shorter template of 2569 nucleotides in length was made by fusing a red fluorescent protein (RFP) gene sequence to the first 321 nucleotides of the replicase protein gene. The RFP fragment was obtained by PCR amplification using forward primer (5′-ATCGCTCGAGATGGTGCTAAGGGCGAAGAG-3′) and reverse primer (5′-CAGTCCGCGGCTAATTAAGTTTGTGCCCCAGTTTG-3′) and PVARFP as a template [36]. The primers add *Xho*I and *Sac*II restriction sites to the RFP fragment. The vector backbone for cloning of RFP along with the 35S promoter, N terminal of the replicase protein, TGB3, and the *nos* terminator were generated using forward primer (5′-CTCGAGACGTCCGCGGATGGAAGTAAATACATATCTCAACGCAATC-3′) and reverse primer (5′-CCGCGGACGTCTCGAGGTTTCTTCTCATGTAGTTTAGCTTTCTGGG-3′) and Tem2 as template. The primers add *Xho*I and *Sac*II restriction sites to the vector backbone. Both the PCR fragments were treated with *Dpn*I, *Xho*I, and *Sac*II restriction digestion enzymes (New England Biolabs Inc.) and purified first via gel purification and later with GeneJET Gel Extraction Kit (Thermo Fisher Scientific) according to the manufacturer’s instructions. The fragments were ligated and transformed into competent DH5α *E. coli* cells. The construct thus created was named Tem8.

### 2.2. Agrobacterium Mediated Infiltration

All the viral and protein constructs in *Agrobacterium* were inoculated in 5 mL of LB + MAK media [Luria Broth + 10 mM MES [2-(*N*-morpholino)ethanesulfonic acid)] pH 7.5; 20 µM acetosyringone + Kanamycin (10 mg/mL)] and incubated overnight at 28 °C. A secondary culture was initiated by inoculating 5 mL fresh LB + MAK media with 200 μL of the primary culture and incubating at 28 °C for 5 h. The cells were harvested by centrifuging the cell culture media at 5000 rpm for 5 min at 20 °C. The harvested cells were washed twice with induction buffer (10 mM MES pH 7.5, 10 mM MgCl and 150 µM acetosyringone). The cells containing the viral and protein expression constructs were then infiltrated into the two upper fully developed *N. benthamiana* leaves using a needle-less syringe at OD_600_ as specified in each experiment.

A firefly luciferase protein expression construct [40] was used in the experiments as a control to normalize for the transformation efficiency and global gene expression.

### 2.3. Renilla Luciferase Assay

The samples were collected from the inoculated leaves at 4 to 6 days post inoculation (DPI) using a round sampler with approximately 4 mm diameter. After freezing the samples in liquid nitrogen, they were ground in 1.5 mL micro centrifuge cuvettes containing six 2 mm steel beads using a Mixer Mill MM 400 (Retsch, Germany) at a frequency of 30 Hz for one and a half minutes. The samples were prepared and the expression level of the Rluc was measured using the Dual-Luciferase Reporter Assay System (Promega, USA), and half the recommended volumes of Luciferase Assay Buffer II and Stop & Glo.

### 2.4. Sodium Dodecyl Sulfate-Polyacrylamide Gel Electrophoresis (SDS-PAGE) and Western Blot

Samples were collected to detect the viral replicase protein from the locally infected leaves at four to six DPI with the various template constructs and the replicase construct. The samples were ground using the Mixer Mill MM 400 (Retsch, Germany) and were further prepared as per standard Western blot procedure. The replicase protein was detected using a polyclonal antibody produced in rabbits against an N-terminal fragment of the replicase [41], anti-replicase (1:5000), and further incubated with secondary antibody anti-rabbit (1:20,000). An X-ray film was developed after treating the membrane with a substrate (Immobilon western chemiluminescent HRP substrate, Millipore Corporation, USA) for 2 min and exposing the film to the membrane for an appropriate time to detect the desired protein band.

### 2.5. RNA Agarose Gel Electrophoresis and Northern Blot

#### 2.5.1. RNA Gel Electrophoresis and Blotting

RNA was isolated from locally inoculated plant samples collected at six DPI using TRIzol (Ambion, Life technologies, USA). Each sample containing 2 μg of RNA was mixed with freshly prepared RNA loading buffer (1:2) and heated at 95 °C for 5 min. The samples were run on a denaturing 1% agarose gel for 1 h at 30 V and 5 h at 70 V. After electrophoresis, the gel was cut into the desired size and stabilized in 20x saline-sodium citrate (SSC) for 10 min. A capillary blot transfer system was assembled to transfer the RNA onto a positively charged Amersham Hybond-N nylon filter (GE Healthcare Life Sciences, UK). The setup was disassembled the next day and RNA was crosslinked to the membrane with ultraviolet (UV) light using Stratalinker (Stratagene, USA). The blot was washed for 5 min with sterile water and stained with methylene blue stain before being washed twice with sterile water for 5 min each.

#### 2.5.2. Probe Synthesis

Rluc and T7 promoter sequences were amplified through PCR using primer pairs (5′-GAAACGGATGATAACTGGTCCGCAGTGG-3′), (5′-ATACATAATACGACTCACTATAGGGATTTCACGAGGCCATGATAATG-3′) and (5′-CTTAATAATACGACTCACTATAGGATGATAACTGGTCCGCAGTGGTG-3′), (5′-GAGGCCATGATAATGTTGGACGACGAACTTCAC-3′) was used for in vitro transcription of the sense and antisense probes labeled with [α-^32^P]-CTP. The transcription mix was incubated at 37 °C for one and half hours followed by DNase (2U) treatment for 15 min. The reaction was stopped by the addition of 20 mM ethylenediaminetetraacetic acid (EDTA) and any unincorporated nucleotide was removed with G-25 spin columns (GE Healthcare). The activity of the probe was measured using Microbeta plate counter (Cerenkov Protocol Program).

#### 2.5.3. Hybridization of the Membrane and Detection of Signal

The membrane was prehybridized with hybridization buffer and incubated at 60 °C for one and half hours. One mL of the plus probe was mixed with the hybridization buffer (1 × 10^6^ counts/mL), boiled for 5 min, added onto the hybridizing membrane and then incubated overnight at 60 °C. A low-stringency wash buffer (2X SSC + 0.1% SDS) and a medium-stringency wash buffer (0.5X SSC + 0.1% SDS) were used to wash the membrane for 15 min each. This was followed by two washes with a high stringency wash buffer (0.1 X SSC + 0.1% SDS) for 15 min. The first three washes were conducted at room temperature, while the last wash was carried out at 65 °C. The membrane was then wrapped in Saranwrap and exposed to an imaging plate inside an X-ray cassette for 72 h. The signal was detected using a variable-mode imager, Typhoon trio (GE Healthcare Life Sciences).

To detect the positive strand RNA, the membrane was stripped off the plus probe twice with stripping buffer (0.01X SSC, 0.5% SDS) at 99 °C and then hybridized again with the minus probe.

### 2.6. Imaging Cells Supporting Trans-Replication

Locally inoculated leaves of *N. benthamiana* with template expressing RFP, Tem8, and the replicase protein expression construct at 6 DPI were visualized under an Axioskop 2 plus microscope (Carl Zeiss, Germany). Digital images were recorded using an AxioCam HRc microscope camera and AxioVision 4.8 software (Carl Zeiss).

### 2.7. Statistical Analysis

Student’s *t*-test was performed using Microsoft Excel (2016) to calculate the significance of differences.

## 3. Results

### 3.1. Trans-Replication of Long and Medium Length Templates

Several template constructs were cloned to test if exogenous expression of PVX replicase protein is able to recognize and initiate *trans-*replication (Figure 1). Long genomic templates Tem1 and Tem2 carried a frame-shift mutation in the replicase encoding sequence to block replicase expression from them (Figure 1). Additionally, the CP coding sequence was deleted from two of the long templates, Tem3 and Tem4, to prevent the formation of virions (Figure 1). The templates Tem5, Tem6, and Tem7 (medium templates) had a considerable part of its replicase coding sequence removed with an *Nco*I endonuclease digestion (Figure 1), but Tem 6 and Tem7 retained similar frame-shift mutation and CP deletion as Tem3 and Tem4, respectively. The octa-nucleotide viral sequences at the 5′- and 3′-UTR and NUEs which had been reported to be important for replication were retained [23,31]. PVX-Rluc served as a replication-competent control.

The *trans-*replication efficiency of PVX was estimated by measuring the expression level of the Rluc gene expressed under a second copy of the viral CP’s sg promoter from the template RNA. The PVX replicase protein consists of 1456 amino acid residues. We removed the viral sequences, including 5′- and 3′-untranslated regions, only retaining the replicase sequence with an engineered reference 5′-UTR (Figure 1; [38]). This was performed to block the recognition of the replicase mRNA by the replicase. The replicase was expressed under the control of the cauliflower mosaic virus 35S promoter (CaMV 35S) and a *nos* terminator. All constructs used in the study were expressed in the leaves of *N benthamiana* through *Agrobacterium*-mediated delivery system.

**Figure 2:**
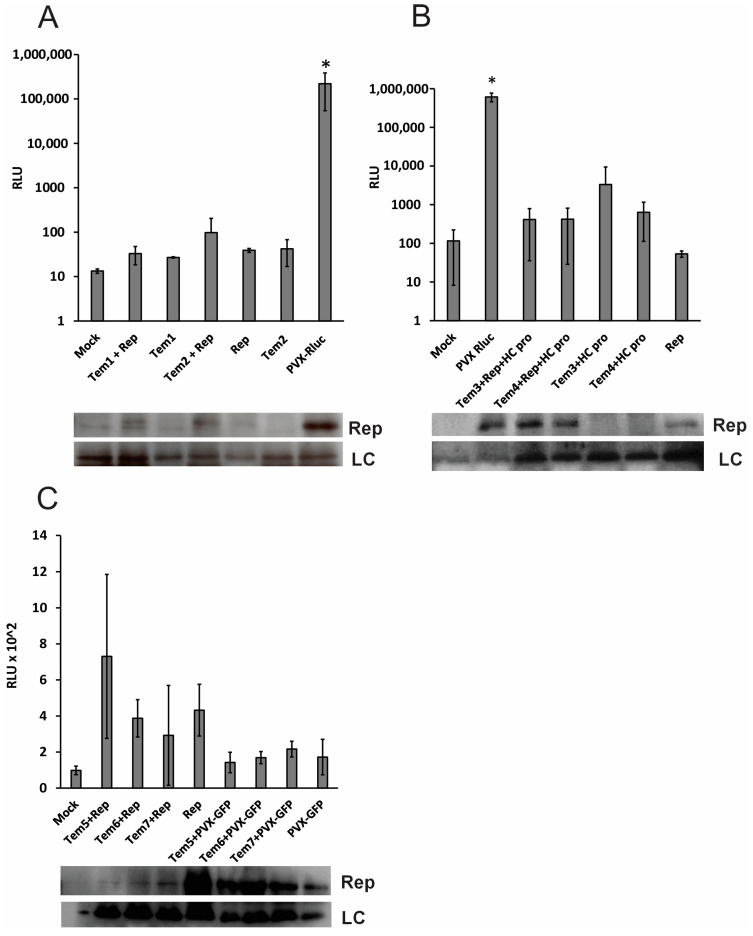
The *trans*-replication efficiency as measured by the dual luciferase assay. Viral gene expression was measured at 4 DPI for the *Renilla luciferase* reporter gene and expression of replicase was detected through Western blot. (**A**,**B**) *Trans-*replication efficiency of the long templates, Tem1 and Tem2; and Tem3 and Tem4 in the presence of HCPro. (**C**) Trans-replication of the medium length templates, Tem 5-Tem7, in the presence of both replicase (rep) and PVX-GFP. (**A**–**C**) Western blot samples corresponding to Rluc panels, showing bands obtained for expression of PVX replicase protein and the loading control (LC). RLU-relative light unit. DPI- days post inoculation. Student’s *t*-test was carried out to estimate the significance of difference between PVX-Rluc versus the mock inoculated sample (* *p* < 0.05).

The long and medium templates (Tem1–7) did not show evidence for replication when the replicase protein was provided in *trans* (Figure 2). The expression of the replicase protein attests to the proper transcription and translation of the protein construct. Dual luciferase assay-derived Rluc gene expression levels were similar to those in mock infiltrated plants, while PVX-Rluc wild type virus yielded Rluc expression levels several fold higher than the mock infiltrated, and template and replicase co-expressed plants (Figure 2A,B). The replicase protein was abundantly produced both from the protein expression construct and the wild type virus, as determined by Western blotting using the polyclonal anti-PVX replicase antibody (Figure 2B,C). The medium templates were also co-expressed with PVX-GFP, a wild-type virus which produces GFP from a duplicated copy of CP’sg promoter (Figure 2C). When the replicase protein was provided *in trans* from PVX-GFP, the assay yielded even lower values, which were similar to the negative controls (Figure 2C). Rluc gene expression level was observed to be several thousand folds lower than that of the PVX-Rluc wild type virus. Deletion or the presence of the coat protein coding sequence from the templates had no effect on virus replication *in trans*.

Helper component proteinase (HCPro), a viral silencing suppressor encoded by potyviruses, is known to enhance the expression of transgenes. It also aids in the accumulation of PVX virus titer when the plants are infected with both the viruses [42]. Thus, we reasoned that expression of HCPro together with the replicase and the template constructs would be able to promote expression of Rluc produced from a sgRNA. However, we observed that expression of HCPro along with the template sequences and the replicase protein was also unable to elevate the gene expression levels of the long templates. This proved that long templates were not replicating well and thus supported our effort to make the templates shorter.

### 3.2. PVX Replicase Recognizes Short Templates and Initiates Their Replication in Trans

**Figure 3:**
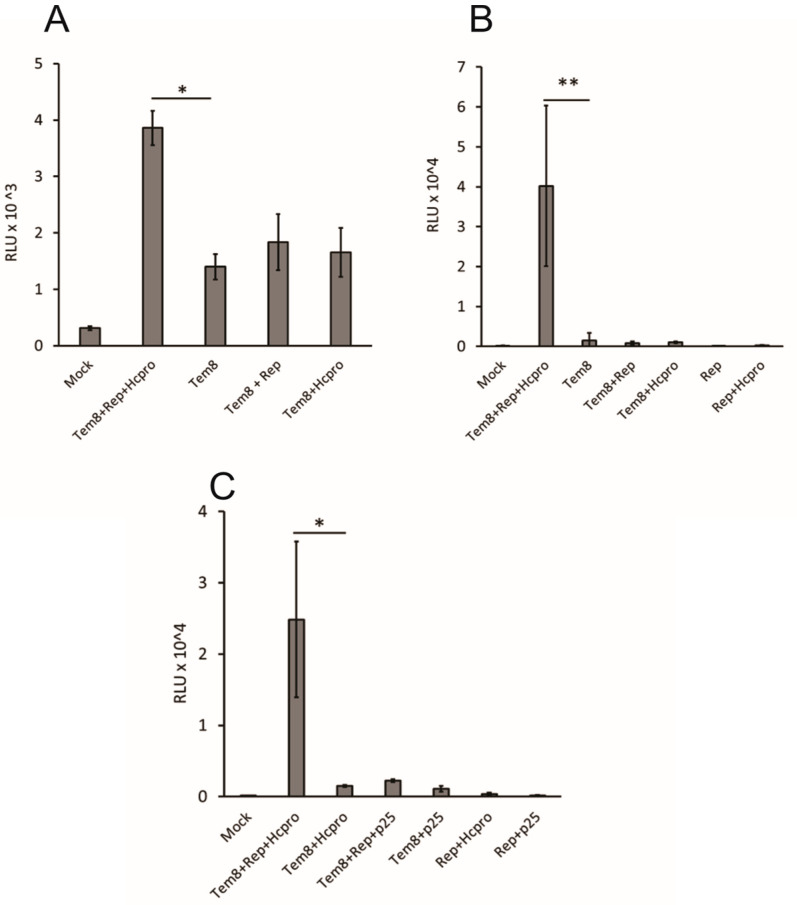
*Trans-*replication of the short templates Tem8 (**A**) at 4 DPI and (**B**) 6 DPI as measured in terms of expression of Rluc gene. (**C**) *Trans-*replication efficiency of Tem8 in the presence of viral silencing suppressors, HCPro and p25. Student’s *t*-test was carried out to estimate the significance of difference between Tem8 co-infiltrated with replicase and HCPro versus Tem8 (**A**,**B**) and Tem8 + HCPro (**C**) (* *p* < 0.05; ** *p* < 0.01).

Previous studies on *trans-*replication of alphaviruses had indicated that shorter templates replicated with a higher efficiency than long templates [34]. Further investigation of the *trans-*replication system with PVX led us to create a short template, Tem8 (Figure 1). An RFP coding sequence was fused to the 5′ 321 nucleotides of the replicase to yield a 39 kDa fusion protein expressed from the genomic CaMV 35S promoter. An Rluc reporter gene was expressed from a duplicated copy of CP sg promoter preceded by the coding sequence of TGB3. All other sequences except for the 5′ and 3′ non-coding regions were eliminated. The resultant template was one-third the size of the long templates and 1.7 times shorter than the medium sized templates. It was agroinfiltrated at OD_600_ 1 with the replicase gene and potyviral HCPro gene at OD_600_ 0.3. The short template, Tem8, replicated when replicase and HCPro were provided *in trans* (Figure 3A,B). The Rluc gene expression levels as measured at 6DPI were 16-fold over the values obtained from the samples inoculated with the templates alone. Samples collected at 4DPI showed a lower replication rate of the templates (Figure 3A), which was further elevated by 6DPI. Following this, samples were collected at 6DPI for all succeeding experiments. Interestingly, agro-infiltration of the template and the replicase in the absence of a silencing suppressor did not support *trans-*replication, as is evident from the declining Rluc values at 6DPI (Figure 3B).

### 3.3. HCPro Supports Trans-Replication of PVX

It was interesting to note that Rluc values, signifying *trans-*replication of PVX, could not be detected in the absence of HCPro. In order to investigate the importance and compatibility of viral silencing suppressors in the *trans-*replication system of PVX, we incorporated p25, PVX encoded viral silencing suppressor, and HCPro with the templates and the replicase. As shown in Figure 3, HCPro supported replication of the templates when the replicase was provided *in trans*. Remarkably, exogenous expression of p25 with the template and replicase was not able to elevate Rluc gene expression and displayed values similar to those plants inoculated with replicase or template alone or the mock inoculated plants (Figure 3C).

### 3.4. Detection of Template Expression by Epifluorescence Microscopy

**Figure 4:**
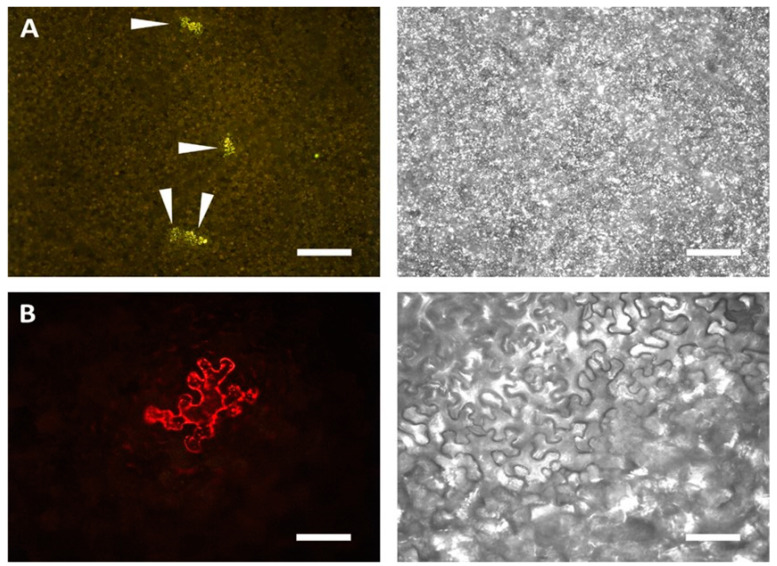
Leaf cells allowing *trans-*replication of PVX. (**A**) Micrograph showing four leaf epidermal cells where replication of short template Tem8 takes place when replicase is provided *in trans*. The arrowheads point at individual fluorescing cells. For better contrast, the RFP signal is presented in yellow. Scale bar 500 μm. (**B**) An individual leaf epidermal cell expressing RFP from the Tem8 construct. Scale bar 100 μm. The images on the right are obtained after the leaf cells have been imaged under white light.

Translation of the genomic strand in Tem8 results in the production of RFP, which imparted the characteristic red color to the leaf cells when viewed under a fluorescence microscope at 546/590 nm excitation/emission wavelength (Figure 4). However, the number of cells exhibiting red fluorescence was scarce and widely scattered across the leaf lamina (Figure 4A), indicating that Tem8 is expressed only in some selected cells. Fluorescence was only observed in leaves transfected with the template and replicase in the presence of HCPro. Leaves transfected in the presence of p25 did not exhibit red fluorescence. This result suggests that expression of HCPro together with Tem8 and Rep stabilizes Tem8. This could be attributed to the fact that HCPro is a stronger silencing suppressor than p25.

### 3.5. Detection of vRNA through Northern Blotting

**Figure 5:**
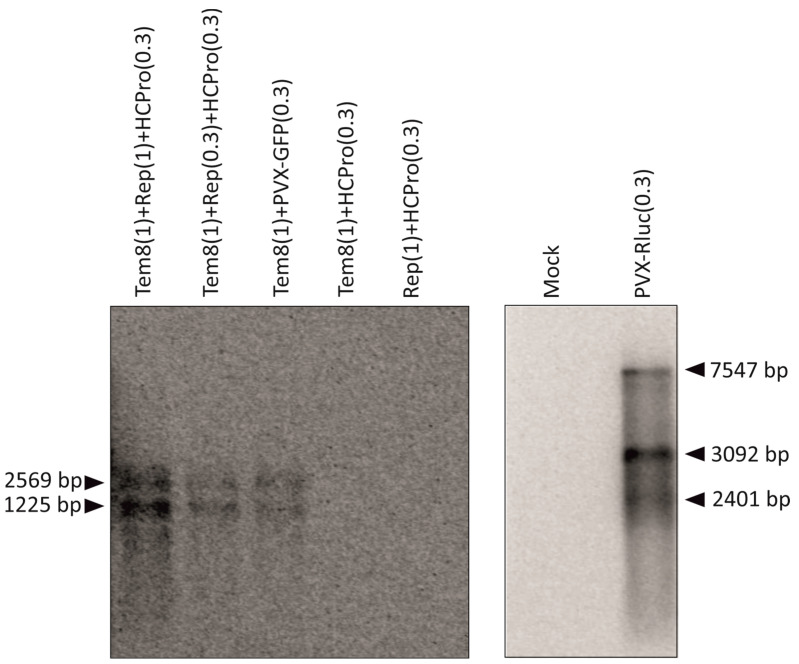
Positive strand genomic and sub-genomic RNA as detected by Northern blotting. Infiltration OD_600_ of the constructs has been indicated within the parentheses. PVX-Rluc and the templates was detected after 24 h and 72 h of exposure, respectively.

As replication of a (+) ssRNA virus results in the production and accumulation of its vRNA, we performed Northern blotting to detect positive strand and the subsequent positive sense sg RNA from samples collected at 6DPI (Figure 5). Tem8 produces one (+)- gRNA of size 2569 bp and a 1225 bp sgRNA, while the PVX-Rluc generates one 7547 bp gRNA and four sgRNAs of size 3092, 2401, 1908, and 839 bp. For PVX-Rluc, we observed three clear bands, one for gRNA and two bands for sgRNA; for the templates co-infiltrated with replicase and HCPro, one band for gRNA and another sgRNA band was observed. The amount of RNA accumulated by 6DPI was highest for Tem8 infiltrated at OD_600_ 1, when replicase was also provided at a similar *Agrobacterium* culture density. *Trans-*replication of the templates in the presence of replicase and HCPro provided from the protein expression construct and PVX-GFP at OD_600_ 0.3 (lanes 4 and 5 respectively, Figure 5) exhibited lower RNA levels with declining band intensity.

## 4. Discussion

We set out to test the feasibility of the *trans-*replication system for PVX *in planta*, and here we report a successful outcome. Short, approximately 1/3 genome length, replication competent templates derived from PVX were able to replicate in the leaves of *N. benthamiana* when the replicase protein was provided *in trans.* However, unlike *trans-*replication systems of alphaviruses in mammalian cells, this system is operational in plants only in the presence of a heterologous silencing suppressor, potyviral encoded HCPro. We proved that the replicase is the only protein required for PVX replication and that it is functional in *trans.* As the short template (Tem8) containing minimum viral sequences is able to replicate, we propose that the deleted sequences are not essential for PVX replication. Nevertheless, they could play a role in replication enhancement and RNA stability.

We have established a functioning *trans*-replication system for PVX. The replicase protein, produced independently from a modified mRNA, is able to recognize the template RNA and replicate it efficiently, albeit in a limited number of cells. As the replicase protein expression construct is developed to prevent self-recognition, and thus replication, by engineering a leader sequence in place of the viral 5′-UTR [38], its production and accumulation in the leaves is found to be at a level lower than that detected from the replicating wild-type virus (Figure 2). However, the quantity of replicase mRNA in the plant cells can be manipulated by infiltrating different concentrations of *Agrobacterium* carrying its expression construct. Thus, further optimization of the *trans*-replication system might provide more robust expression of the replicase.

As mentioned, we observed *trans-*replication in only a small number of cells. We state this because we were able to detect very few strongly RFP-expressing cells in the *N. benthamiana* leaves by epifluorescence microscopy. The enzyme-based detection assay of Rluc, which is produced from sg promoter in our constructs, is highly proficient in detecting the signal obtained from a few cells which supported *trans-*replication, when transfected with both the short template (Tem8) and replicase in the presence of HCPro (Figure 3 and Figure 4). The role of plant defense machinery during *trans-*replication of virus-derived templates cannot be disregarded. A possible reason for the *trans-*replication system to initiate with such a low efficiency could be that the template mRNA is actively degraded by the plant anti-viral silencing system, making it unavailable for the replicase protein to replicate it. The templates which escape or are spared by the defense related proteins remain accessible to the replicase to initiate *trans-*replication in a few scattered cells across the leaf lamina.

Virus-encoded silencing suppressors are essential in preventing active degradation of viral genomes by the host’s anti-viral defenses and proliferate infection in the plant cells [42,43]. It is interesting to note that, in the absence of a viral silencing suppressor, *trans-*replication of PVX failed *in planta*. The *trans-*replication system heavily relies on the protective capability of HCPro against the plant defense related proteins to drive the replication of the templates *in trans*. PVX cognate silencing suppressor p25 was not able to support and sustain the *trans-*replication system, arguing for its incompetence when applied in this system. HCPro undeniably plays a crucial role, as Rluc values could not be detected from leaves co-infiltrated with replicase and Tem8 alone. Addition of HCPro into the system causes disruption of the methionine cycle [36], which might lead to enhanced Tem8 gene expression. We are uncertain if the part played by HCPro in the *trans-*replication system with PVX is due to its antiviral silencing suppressor activity or if it is due to synergistic interaction with the PVX-derived template or the replicase. Although p25 has been reported to be essential for synergism to occur, we consider if a synergistic interaction is to take place in our system, either replicase or the viral UTRs should play a part in it. However, the dynamics of the role of HCPro in this system are yet to be investigated.

*Trans-*replication of the short template indicates that the deleted viral sequences are not necessary for PVX replication. The viral 5′- and 3′-UTRs reported to be essential for PVX replication [23,24,26,27,31] have been retained. The 3′-UTR contains an octa-nucleotide sequence to which host proteins bind and any mutation in it eliminates virus replication [31]. Kim and Hemenway (1996 and 1999) have shown the importance of the 5′- and 3′-UTRs in synthesis and accumulation of PVX plus and minus strands. However, the eliminated sequences from the short template could nevertheless be involved in augmenting the pace of viral replication.

What is remarkable is that replication of the long (Tem1–Tem4) and medium templates (Tem5–Tem7) could not be detected during the experimental timespan, when replicase was provided *in trans*. Since the stability of the templates was not tested, the failure to detect *trans-*replication of these templates could be attributed to several factors. They could have been efficiently degraded by the host silencing machinery, since viRNA is often a good template for RNA interference. On the other hand, the inability to detect higher Rluc values from the long and medium length templates could be because of the low turnover of the replicating templates due to poor initiation of *trans-*replication. Delayed sample collection beyond 6 DPI was not possible since the locally infiltrated leaves either become too old to support *trans-*replication or die because of hypersensitive reaction. Since the templates were movement deficient, the possibility of verifying their *trans-*replication at later time points in the systemic leaves is implausible.

Alphaviruses, such as SFV, chikungunya virus, and sindbis virus have previously been reported to *trans-*replicate in mammalian cells when the replicase polyprotein was provided from a separate RNA [34,35,44,45,46]. Valuable information about the alphavirus replication cycle along with the complex interactions between the virus and the host has been obtained from these *trans-*replication studies [34,35,44,45,46]. Structural requirements for alphavirus replication along with host membrane modifications essential to support viral replication complex formation have been explored in these studies. *Trans-*replication as a strategy has also been explored for the development of vaccines and has been suggested to be a safer and cheaper alternative to traditional RNA-based vaccines [47]. In spite of the fact that PVX has been studied extensively, various keys steps in its replication cycle remain a mystery. With the establishment of the *trans*-replication system, the cell biology and the different processes associated with PVX infection cycle can be investigated in a flexible manner. The mode of initiation of replication complexes and their locations is still unknown. Two movement proteins, TGBp2 and TGBp3, are shown to induce membrane vesicles, but their involvement in replication or transport of the virus is currently ambiguous. Aided with the manipulating ability that the *trans-*replication system provides, both viral and host–cell processes can be examined with a higher degree of experimental freedom.

## Data Availability

The datasets generated and/or analyzed during the current study is available upon reasonable request to the corresponding author.
